# Supplementary material for: Germline-Specific Repetitive Elements in Programmatically Eliminated Chromosomes of the Sea Lamprey (Petromyzon marinus)
Source: Genes (Basel). 2019 Oct 22;10(10):832. doi: 10.3390/genes10100832 (PMC6826781; doi:10.3390/genes10100832)
Supplement: Supplementary file 1 [file genes-10-00832-s001.zip › Fig. S5.pdf]

Figure S5

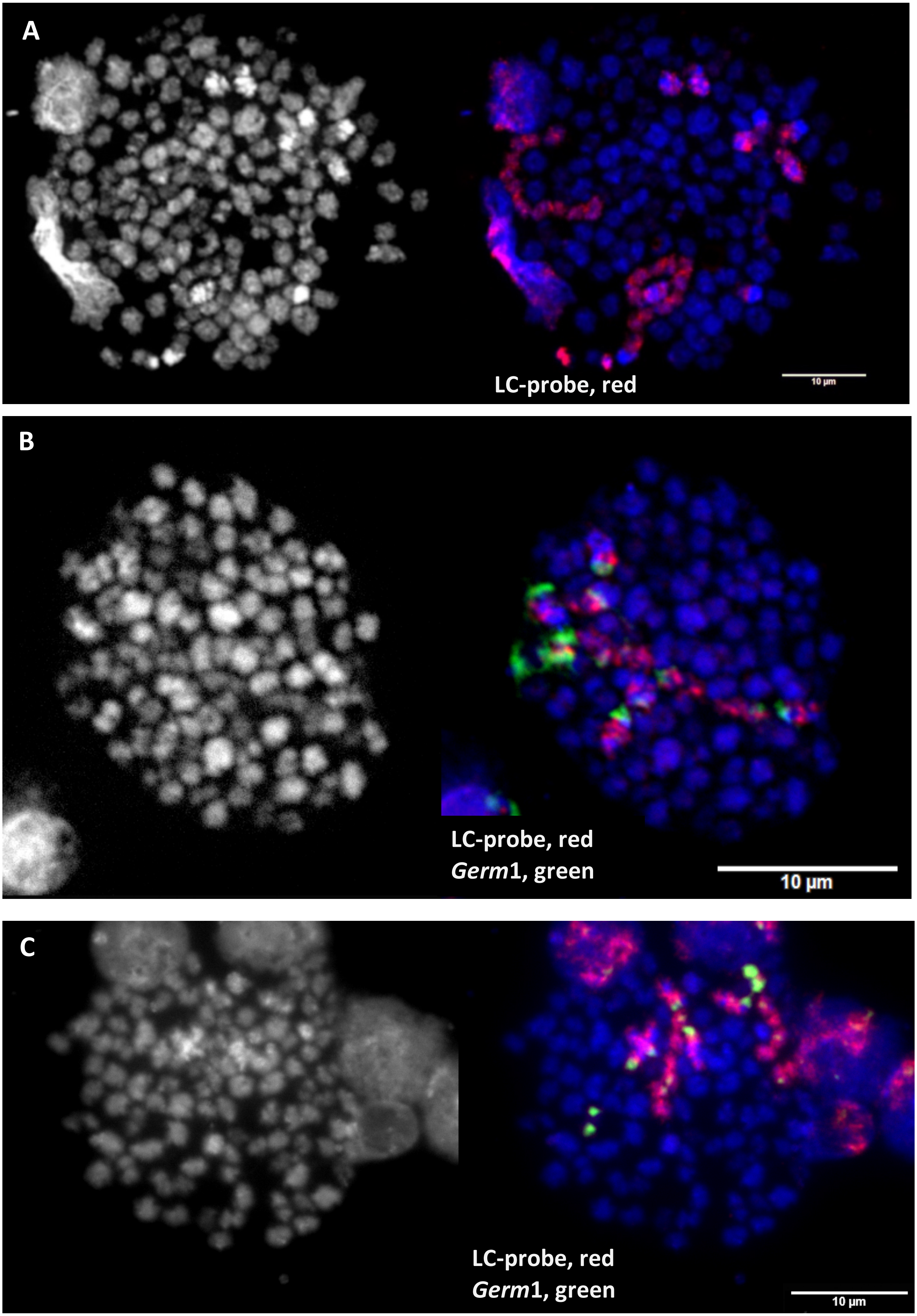
A
LC-probe, red
10 µm
B
LC-probe, red
*Germ*1, green
10 µm
C
LC-probe, red
*Germ*1, green
10 µm

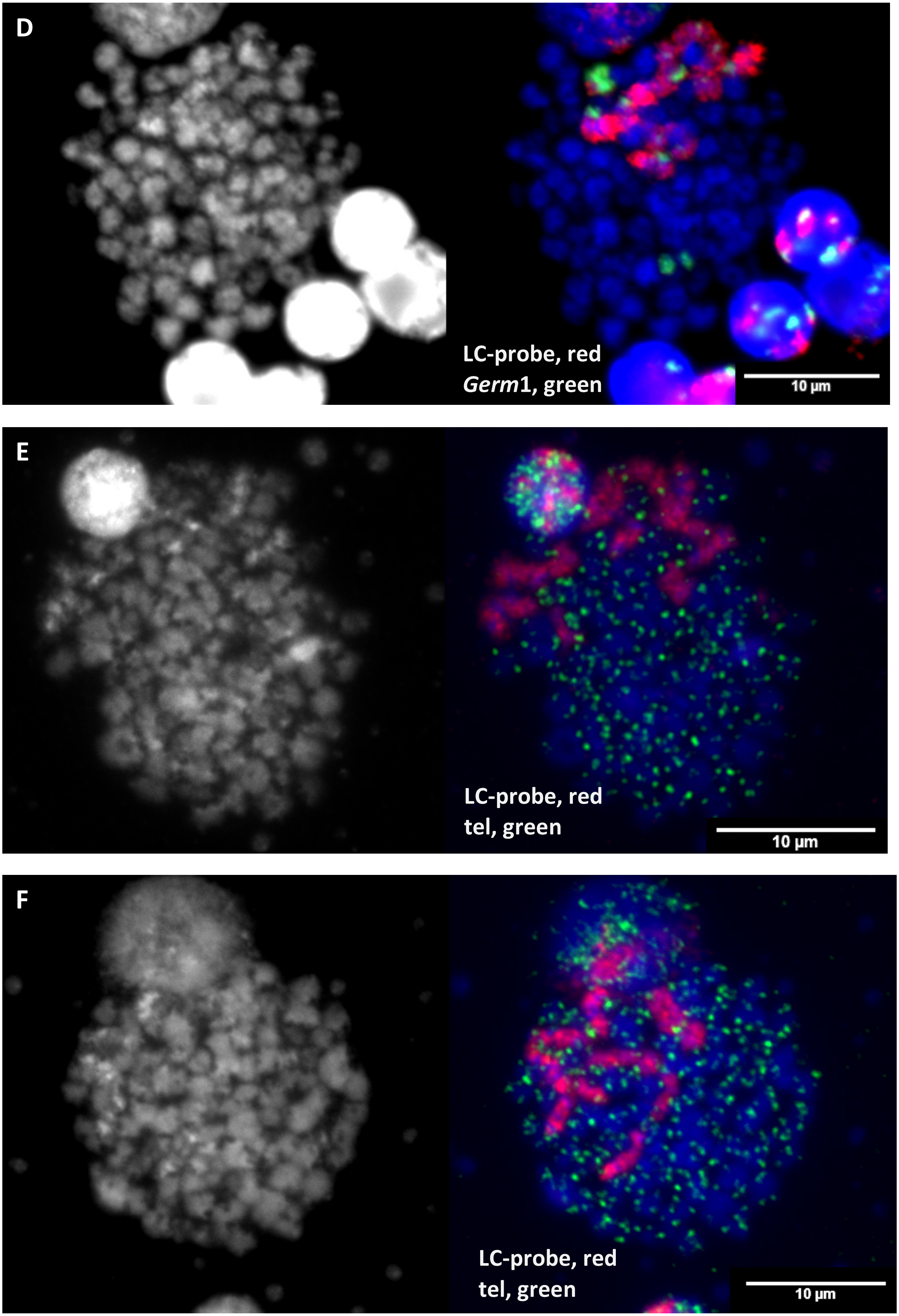
D
LC-probe, red
*Germ*1, green
10 µm
E
LC-probe, red
tel, green
10 µm
F
LC-probe, red
tel, green
10 µm

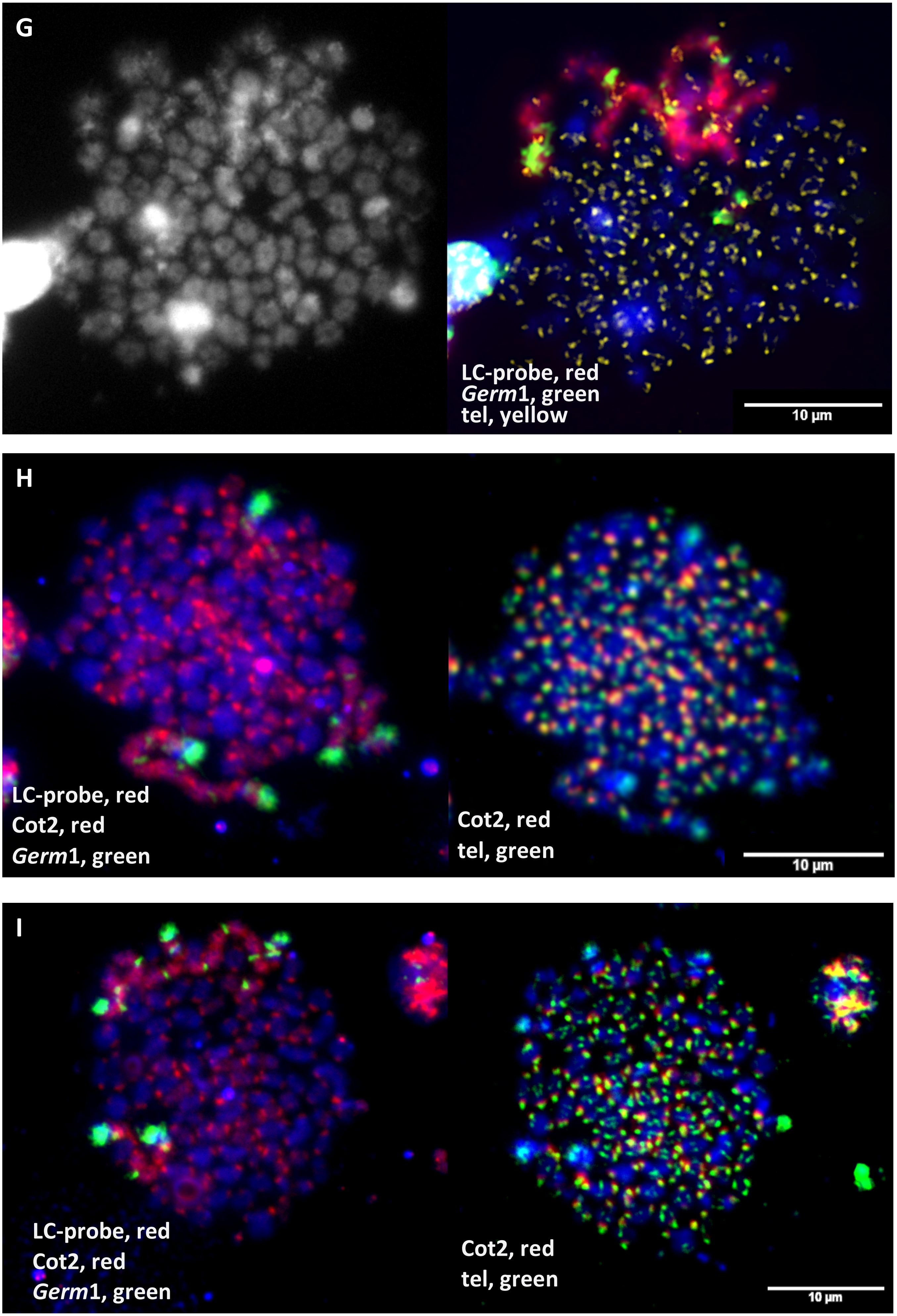
G
LC-probe, red
Germ1, green
tel, yellow
10 µm
H
LC-probe, red
Cot2, red
Germ1, green
Cot2, red
tel, green
10 µm
I
LC-probe, red
Cot2, red
Germ1, green
Cot2, red
tel, green
10 µm

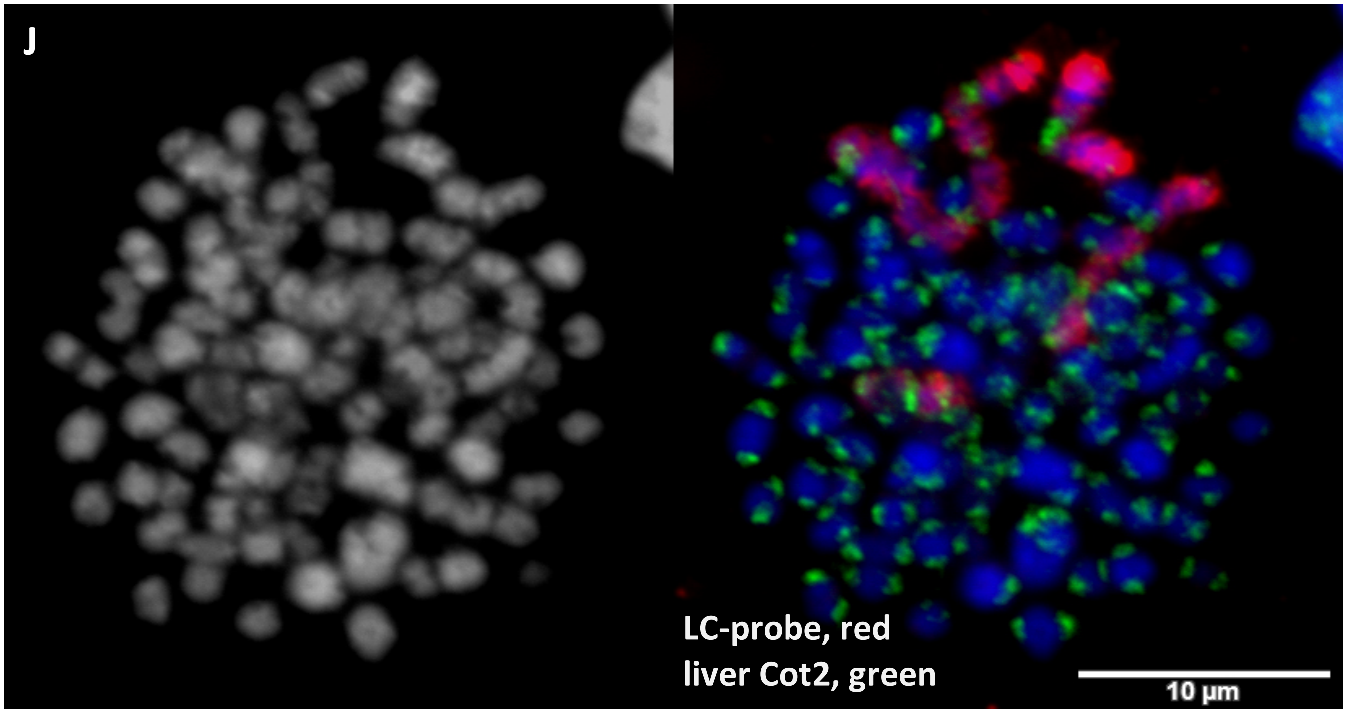
J
LC-probe, red
liver Cot2, green
10 µm

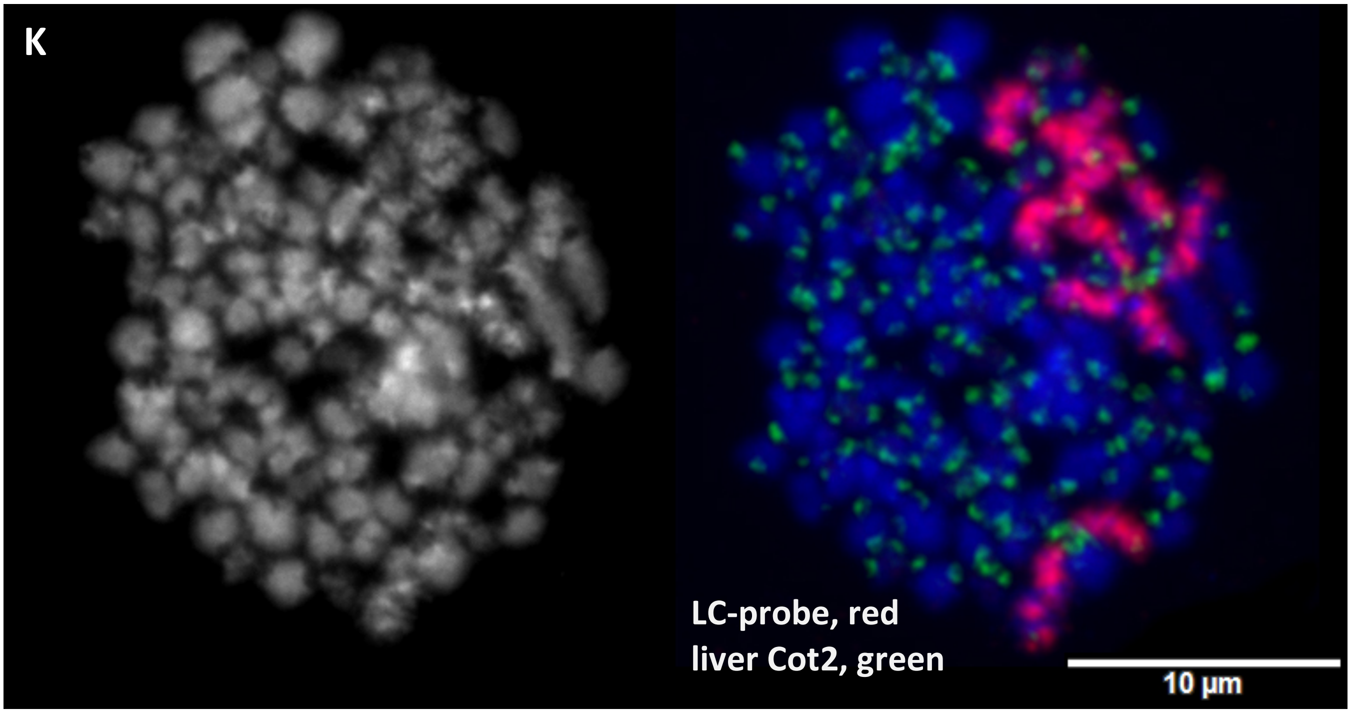
K
LC-probe, red
liver Cot2, green
10 µm

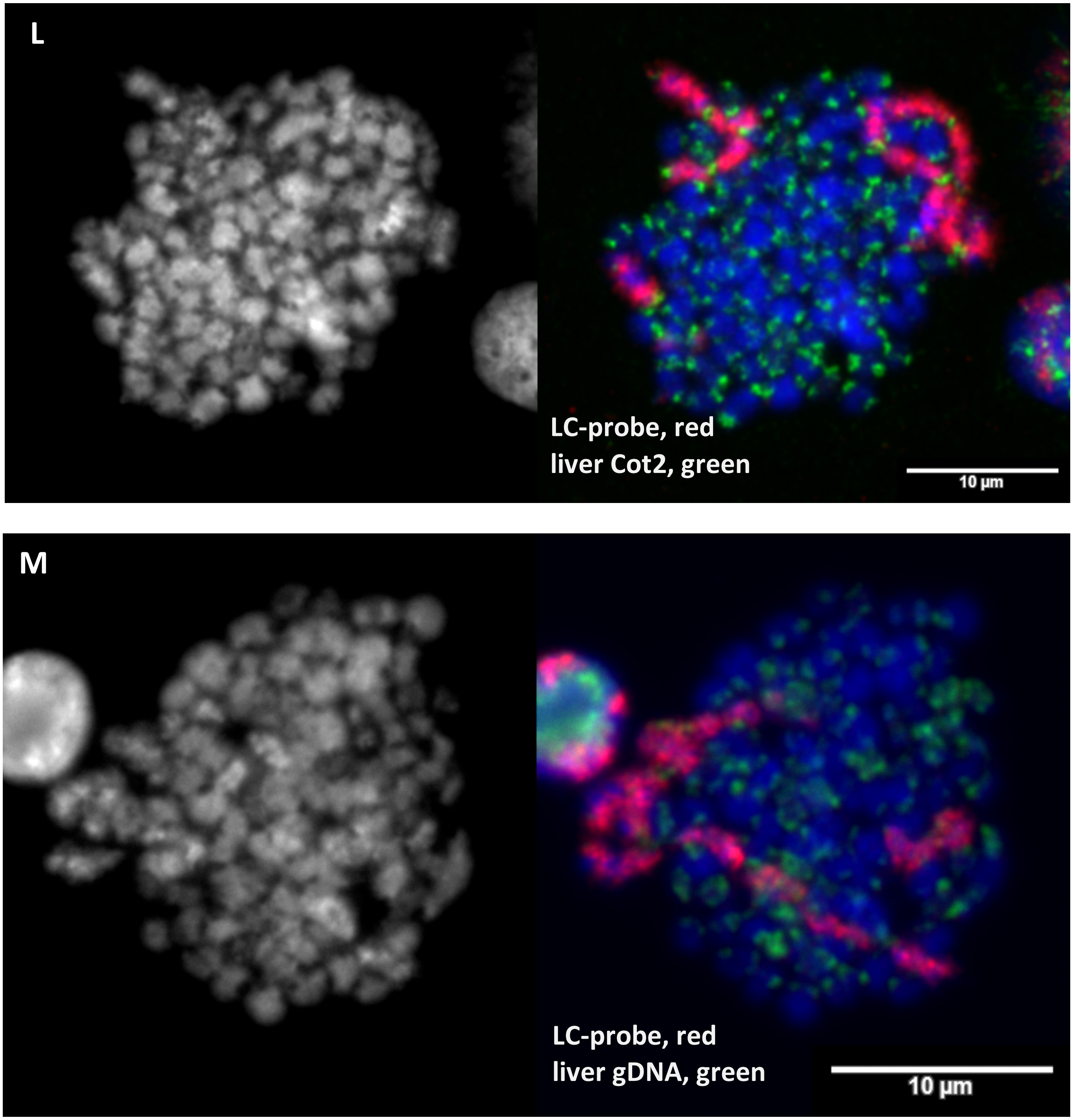
L
LC-probe, red
liver Cot2, green
10 µm
M
LC-probe, red
liver gDNA, green
10 µm

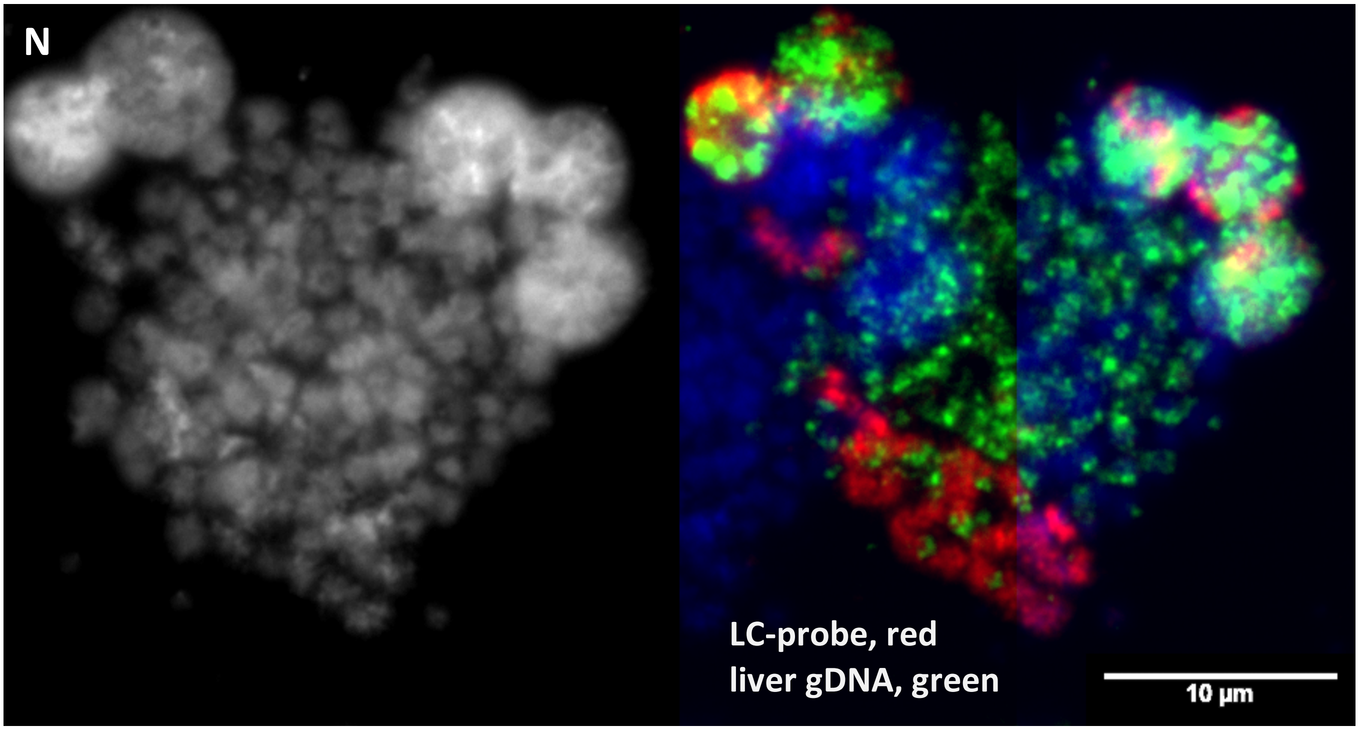

Figure S5. Meiotic chained multivalents in the sea lamprey spermatid metaphase spreads. Meiotic chains in the sea lamprey metaphase I spreads (MI) hybridized with the LC probe in combination with various other probes. (A) LC probe only (red); (B – D) LC (red) and *Germ*1 (green); (E, F) LC (red), telomere PNA probe (green); (G) LC (red), *Germ*1 (green), telomere PNA probe (yellow); (H, I) left: LC (red), testes C_0t2 (red), *Germ*1 (green); right: testes C_0t2 (red), telomere PNA probe (green); (J-L) LC (red), liver C_0t2 (green); (M, N) LC (red), genomic liver (green).
