# Supplementary material for: Germline-Specific Repetitive Elements in Programmatically Eliminated Chromosomes of the Sea Lamprey (Petromyzon marinus)
Source: Genes (Basel). 2019 Oct 22;10(10):832. doi: 10.3390/genes10100832 (PMC6826781; doi:10.3390/genes10100832)
Supplement: Supplementary file 1 [file genes-10-00832-s001.zip › Fig. S4.pdf]

Figure S4

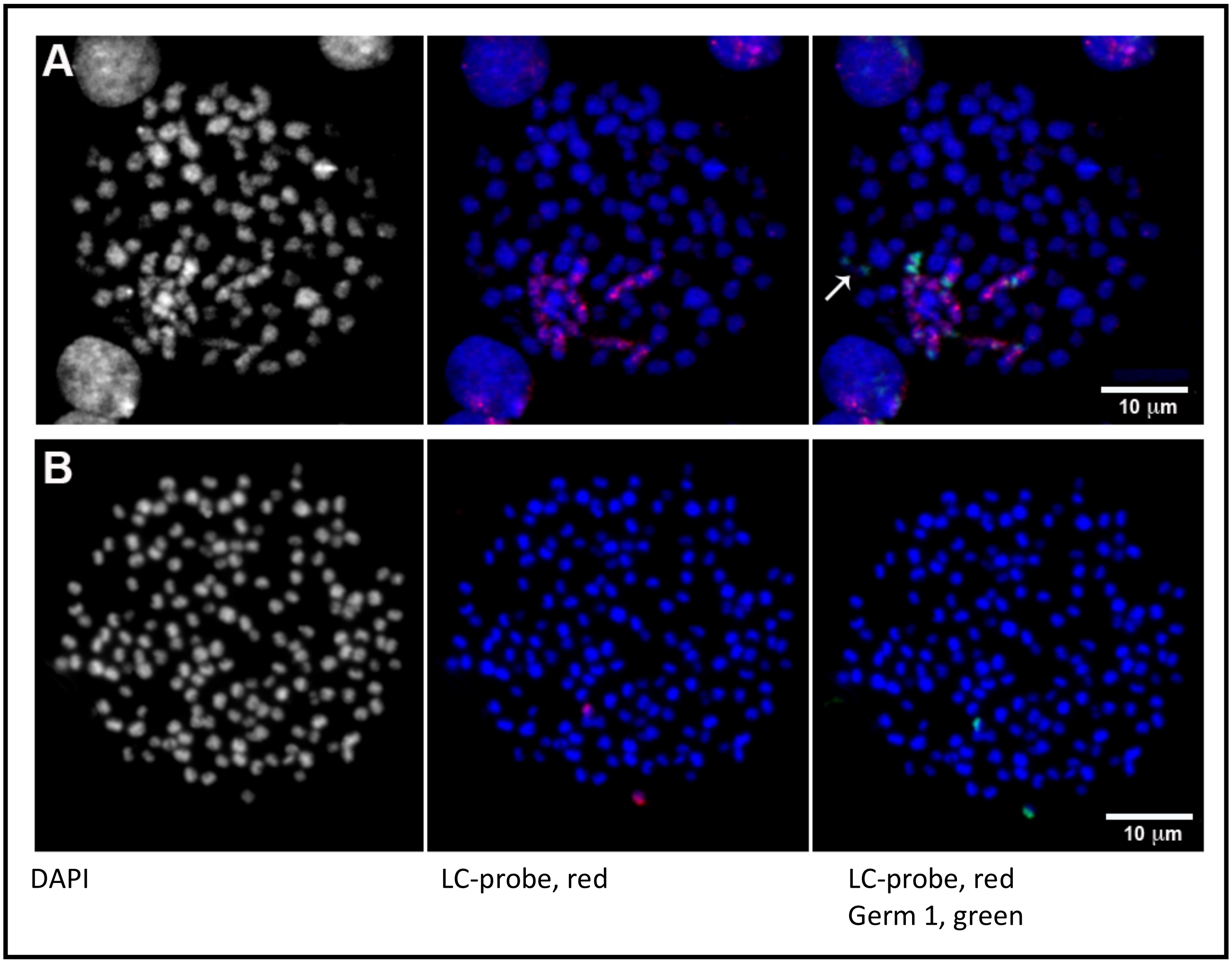

Figure S4. FISH of a probe generated from laser capture of lagging chromatin (red) and *Germ1*-repeat (green) on meiotic (germline) and mitotic (somatic) chromosomes from somatic line. (A) Hybridization to meiotic chromosomes from testes**.** The arrow on panel A indicates bivalent that corresponds somatically retained *Germ*1 enriched chromosome visible in somatic spreads. (B) A somatic metaphase spread contains one pair of chromosomes which shows hybridization both for LC probe and Germ1.
