# Supplementary material for: Germline-Specific Repetitive Elements in Programmatically Eliminated Chromosomes of the Sea Lamprey (Petromyzon marinus)
Source: Genes (Basel). 2019 Oct 22;10(10):832. doi: 10.3390/genes10100832 (PMC6826781; doi:10.3390/genes10100832)
Supplement: Supplementary file 1 [file genes-10-00832-s001.zip › Fig. S6.pdf]

Figure S6

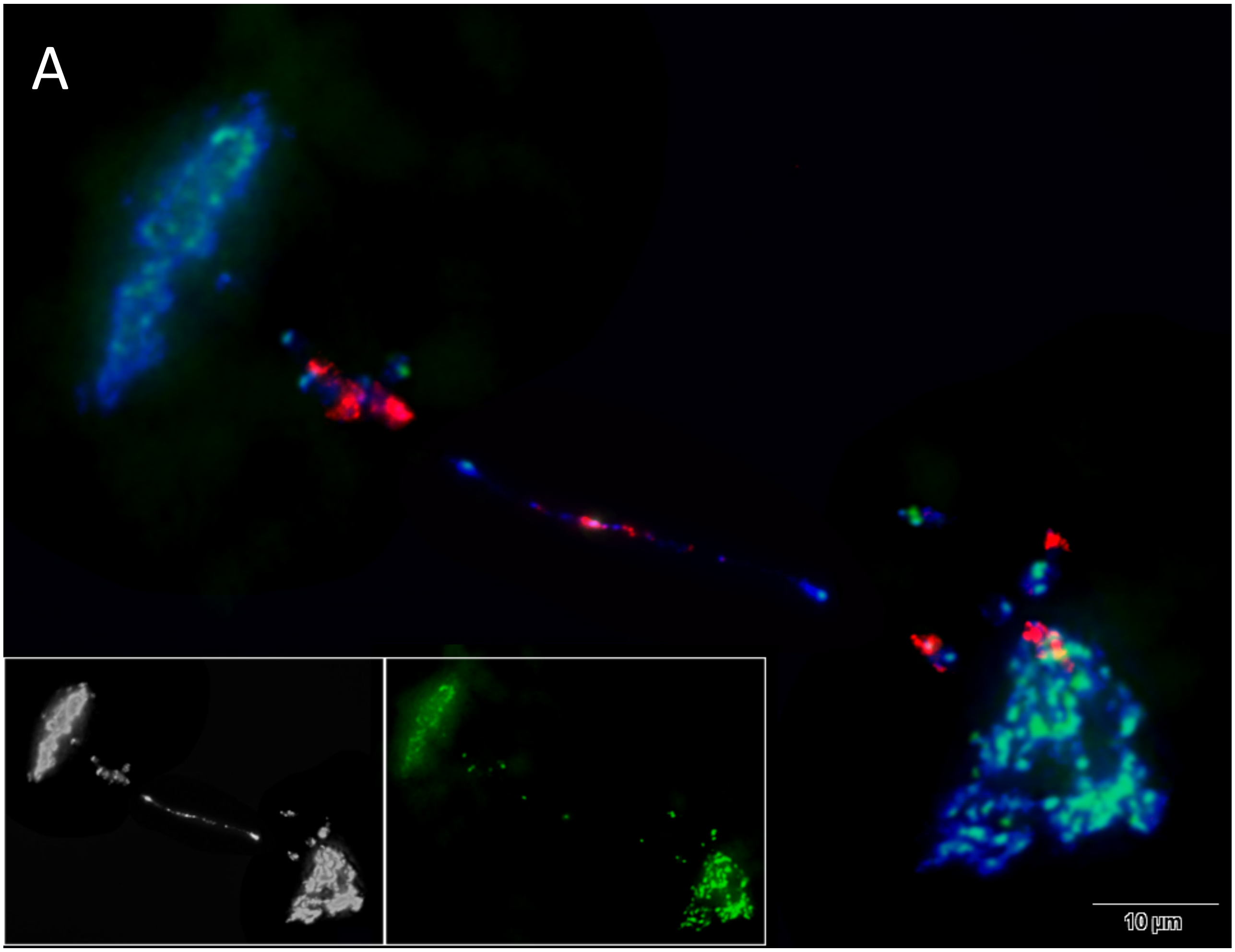

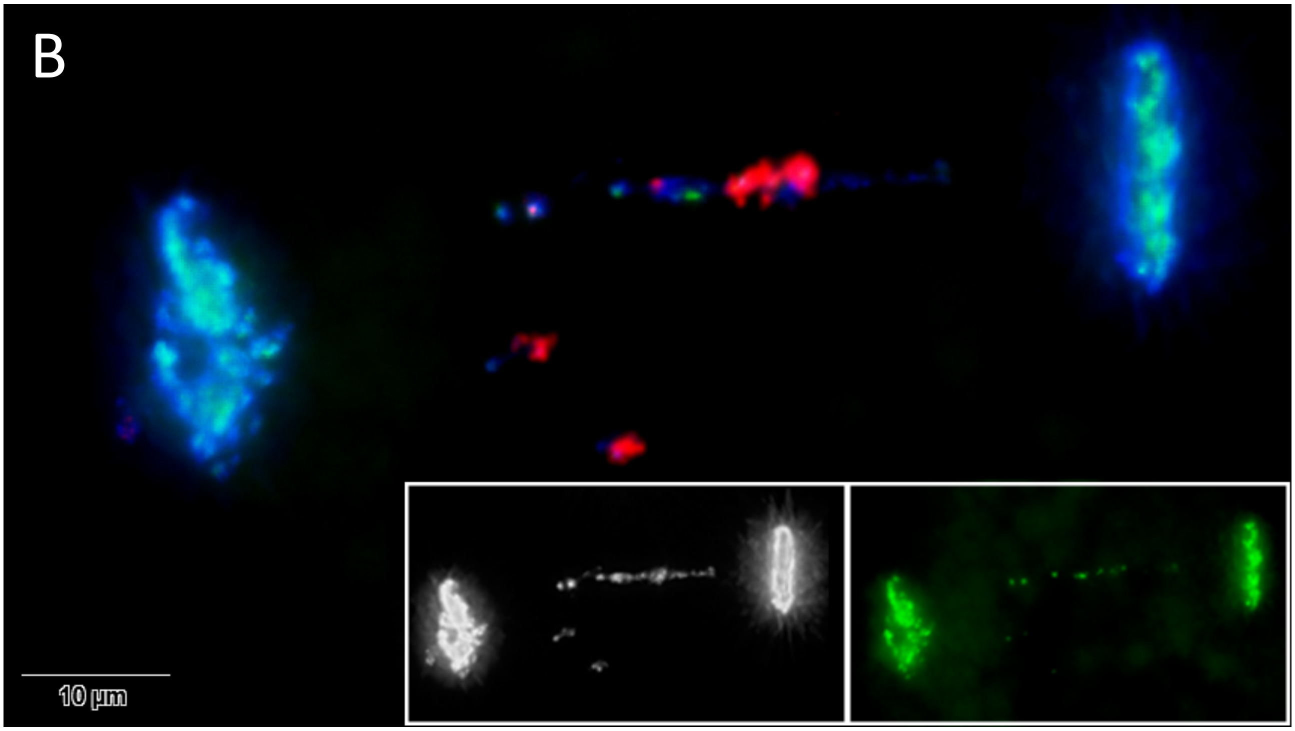

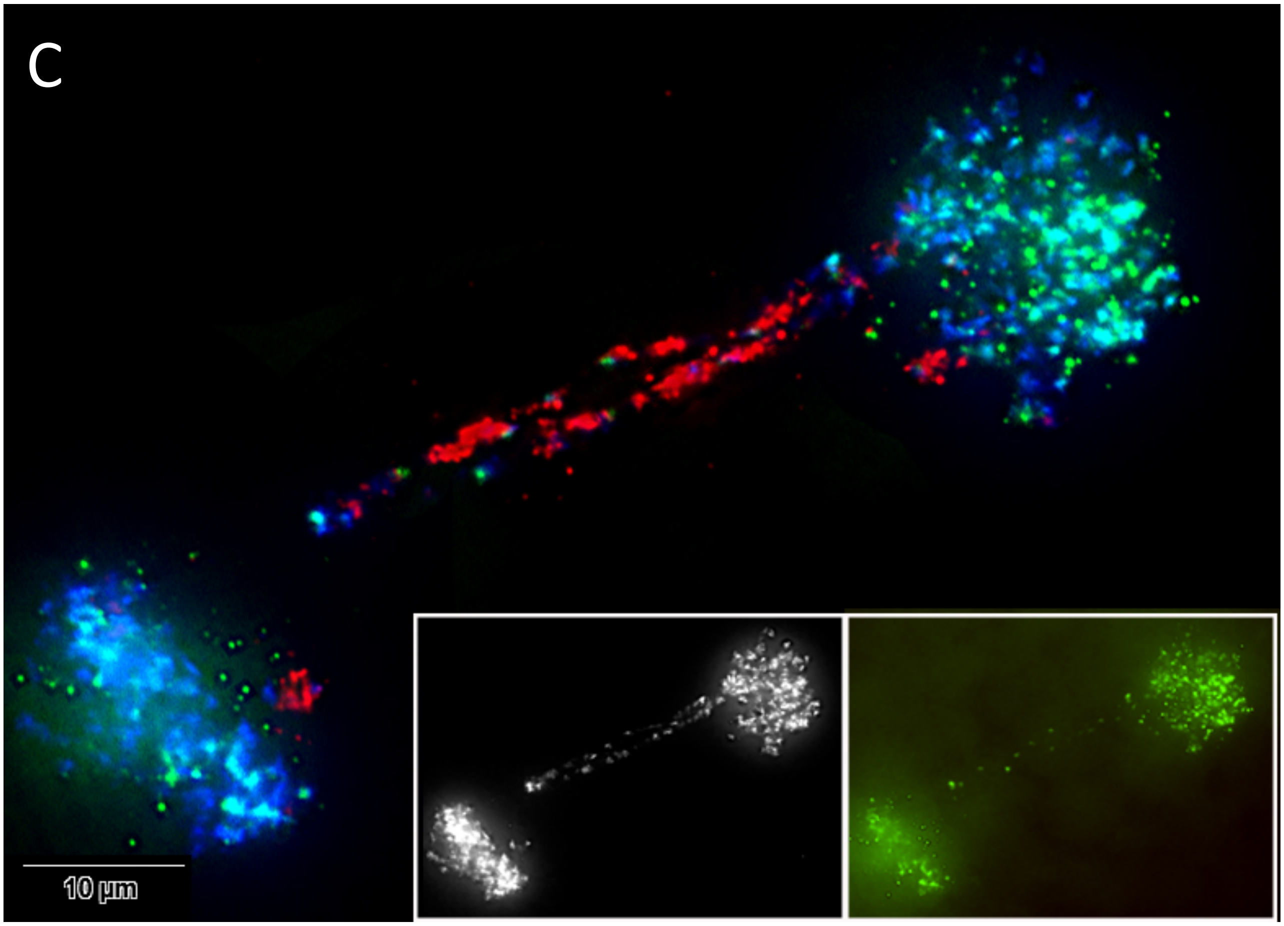
C
10 µm

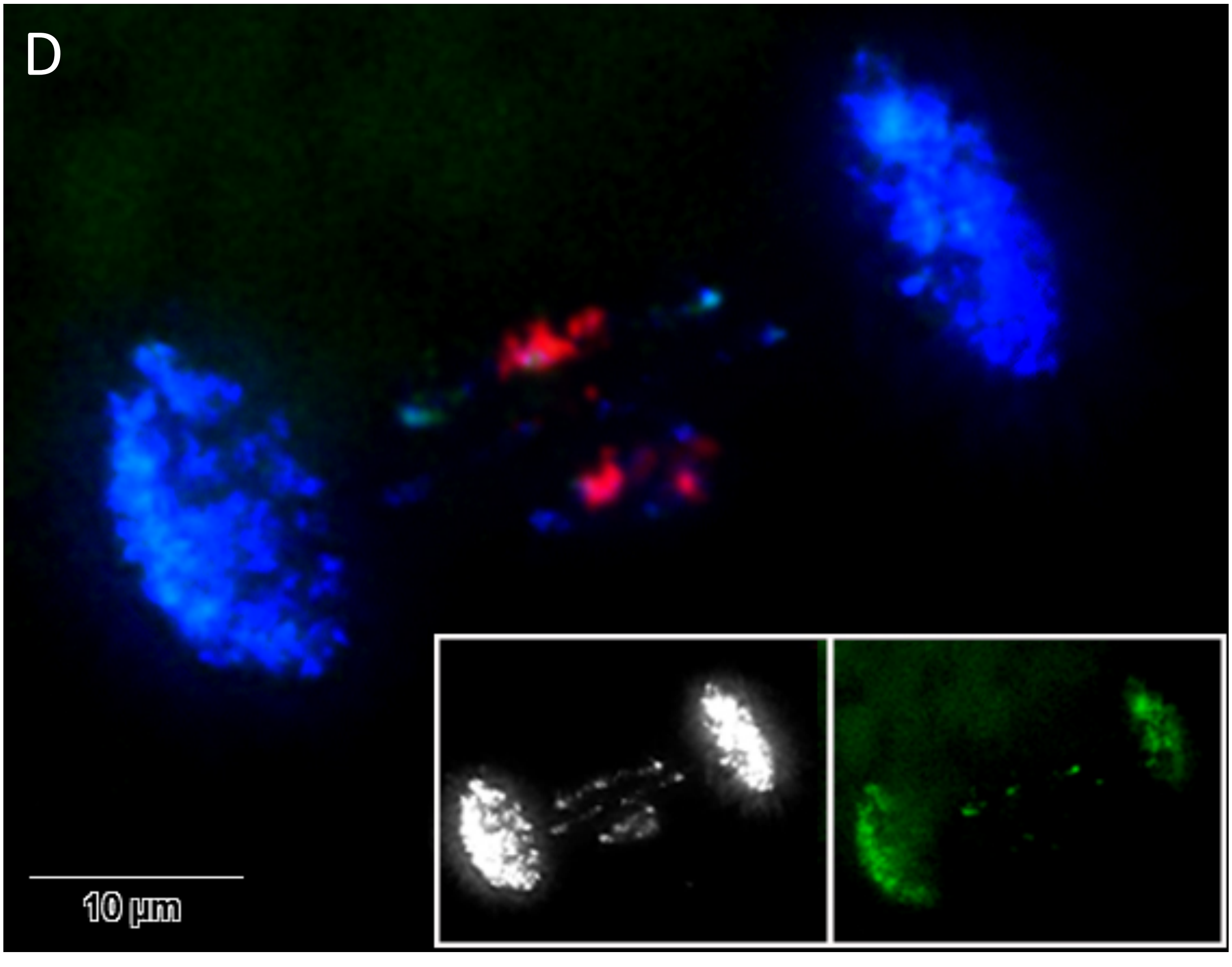
D
10 µm

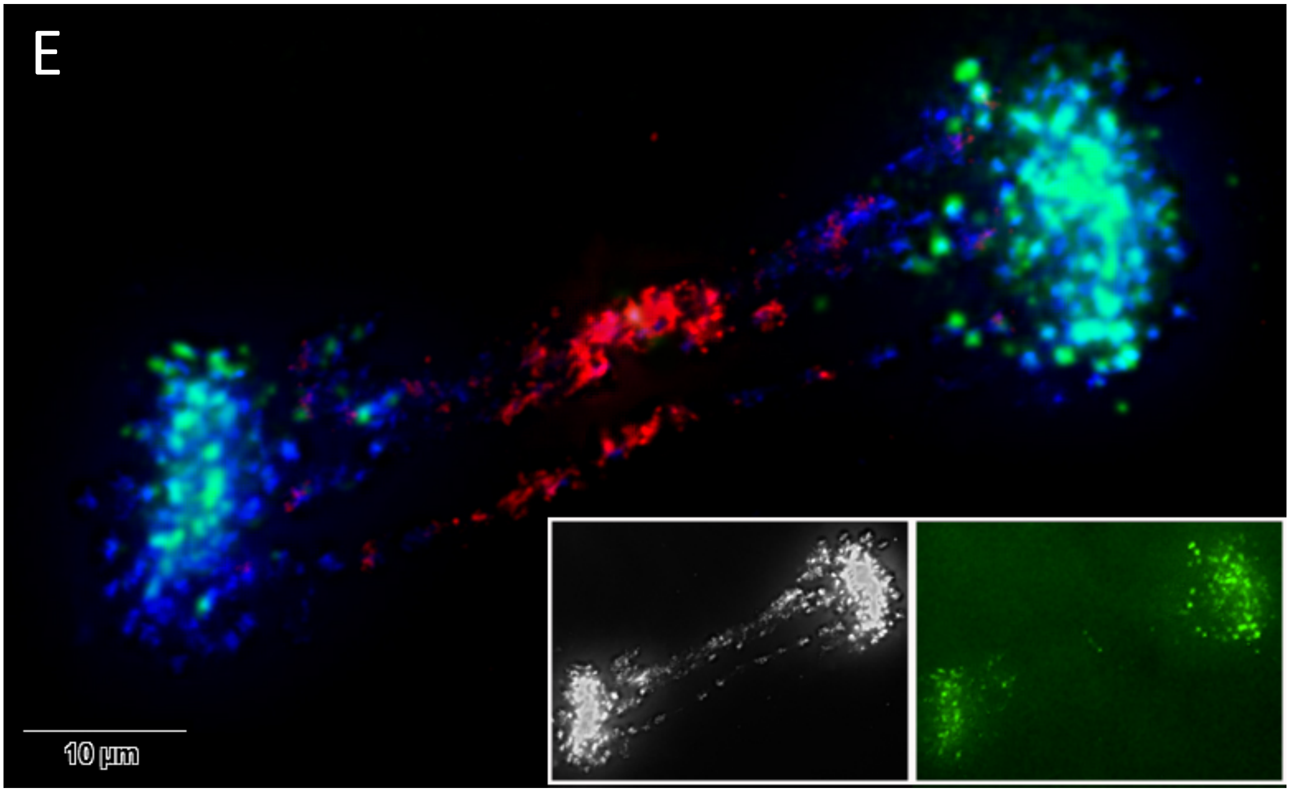
E
10 µm

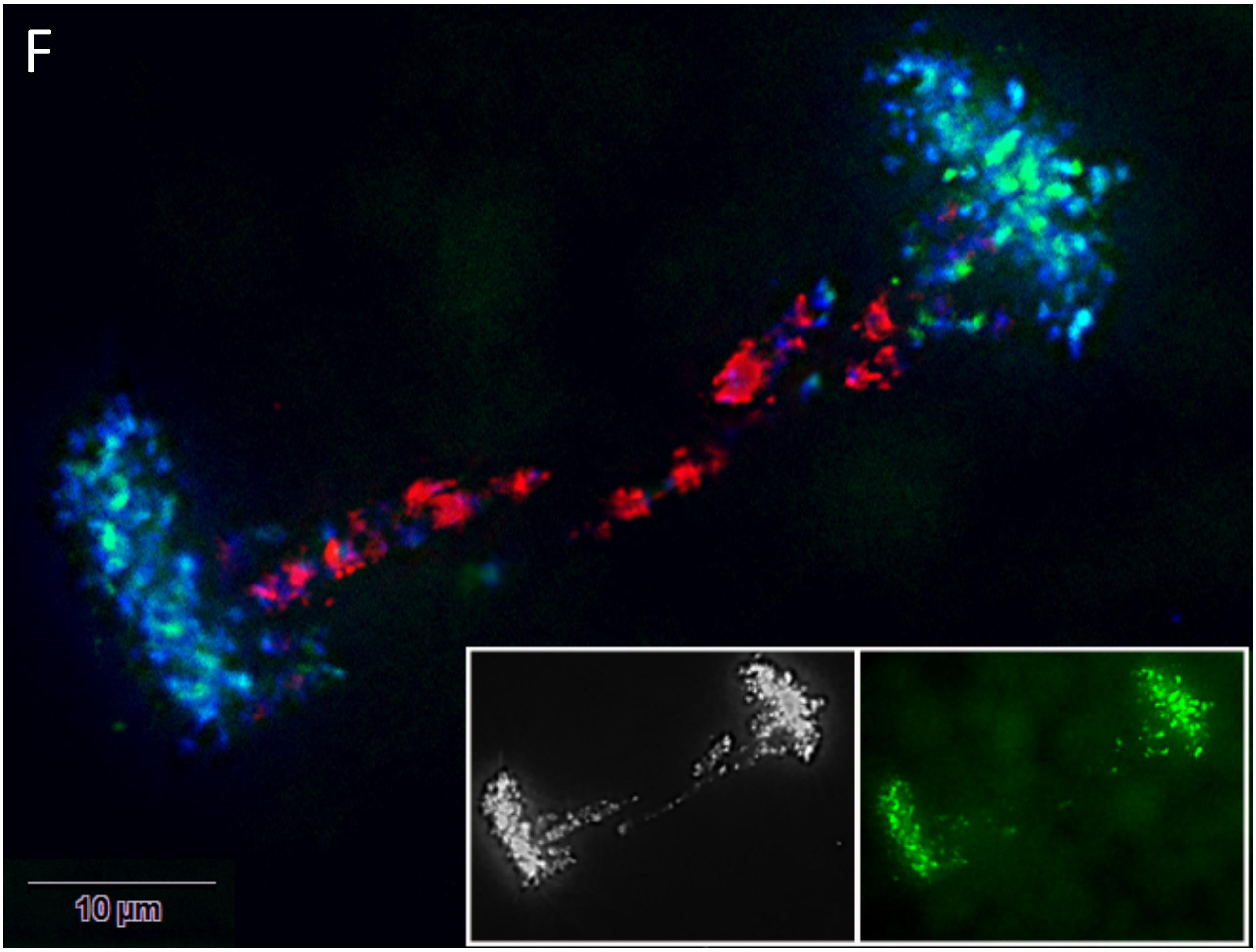
F
10 µm

Figure S6. Telomere dynamics in lagging anaphases. Fluorescence *in situ* hybridization of the *Germ*2 probe (red), a PNA-probe for telomeric repeats (green), and testes genomic DNA (Cy5 labeled, pseudocolored in blue) to eliminating anaphases of 1.5 dpf sea lamprey embryos. Separate counterstain grayscale and green channels are also shown in an additional white framed panel on each image. Stretched chromosomes typically have telomeric signals visible on both ends, equatorial telomeric signals appear as both merged (A, B, C, D, E) and split (C, F) signals.
