# Supplementary material for: Germline-Specific Repetitive Elements in Programmatically Eliminated Chromosomes of the Sea Lamprey (Petromyzon marinus)
Source: Genes (Basel). 2019 Oct 22;10(10):832. doi: 10.3390/genes10100832 (PMC6826781; doi:10.3390/genes10100832)
Supplement: Supplementary file 1 [file genes-10-00832-s001.zip › Fig. S3.pdf]

Figure S3

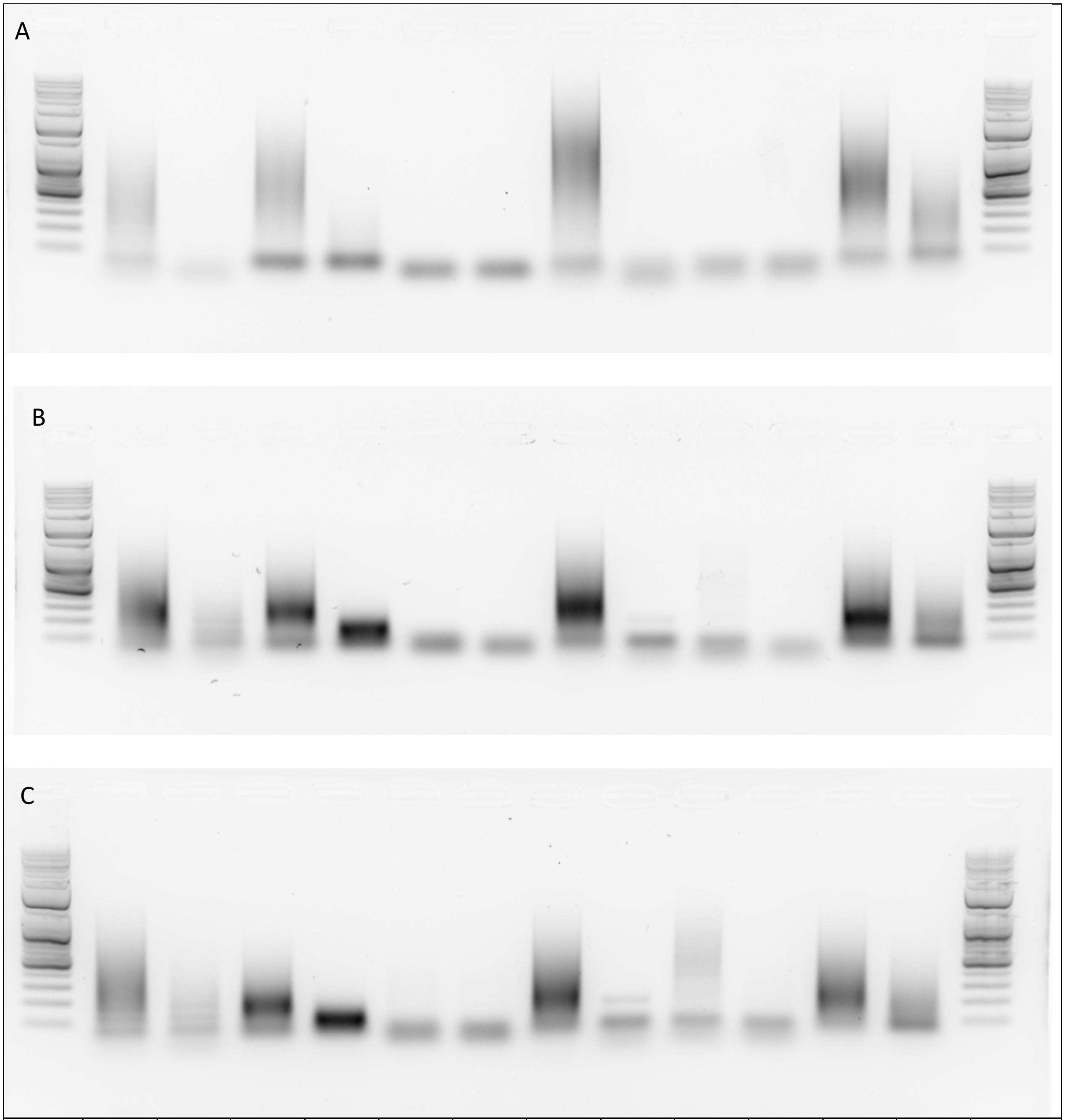

100 Bp ladder	T	fM	T	fM	T	fM	T	fM	T	fM	T	fM	100 bp ladder
	Germ 2		Germ 3		Germ 4		Germ 5		Germ 6		Germ 7		

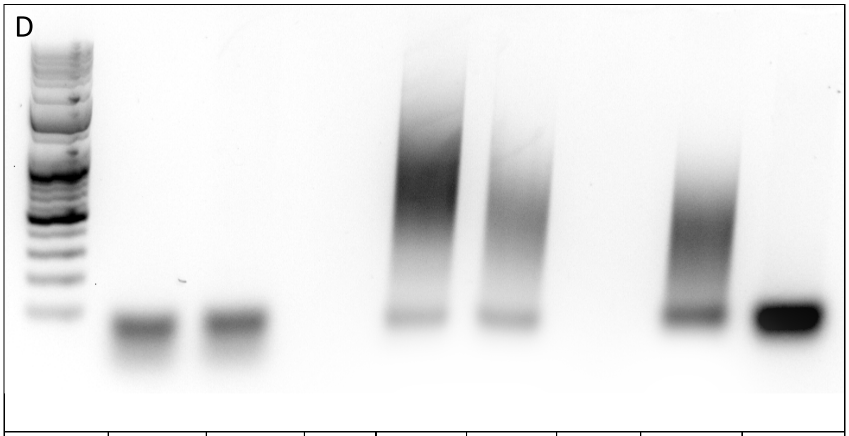

100 Bp ladder	T	fM		T	fM		T	fM
	Rep 13			Germ6			Germ4	

Figure S3. Validation of germline enriched repeats via PCR. DNA extracted from testes ‘T’ was used as a germline sample, and DNA extracted from female muscle tissue as a somatic sample with reduced potential for germline contamination ‘fM’ [69]. Panels A-D show different levels of specificity of PCR reactions depending on the primer annealing temperature: (A) 65 ^{o}C, (B) 60 ^{o}C, (C) 55 ^{o}C, (D) An additional 50 ^{o}C PCR gel was run for repeat13 (not used in further analysis), *Germ*6, and *Germ*4.
