# Supplementary material for: Germline-Specific Repetitive Elements in Programmatically Eliminated Chromosomes of the Sea Lamprey (Petromyzon marinus)
Source: Genes (Basel). 2019 Oct 22;10(10):832. doi: 10.3390/genes10100832 (PMC6826781; doi:10.3390/genes10100832)
Supplement: Supplementary file 1 [file genes-10-00832-s001.zip › Fig. S2.pdf]

Figure S2

A.

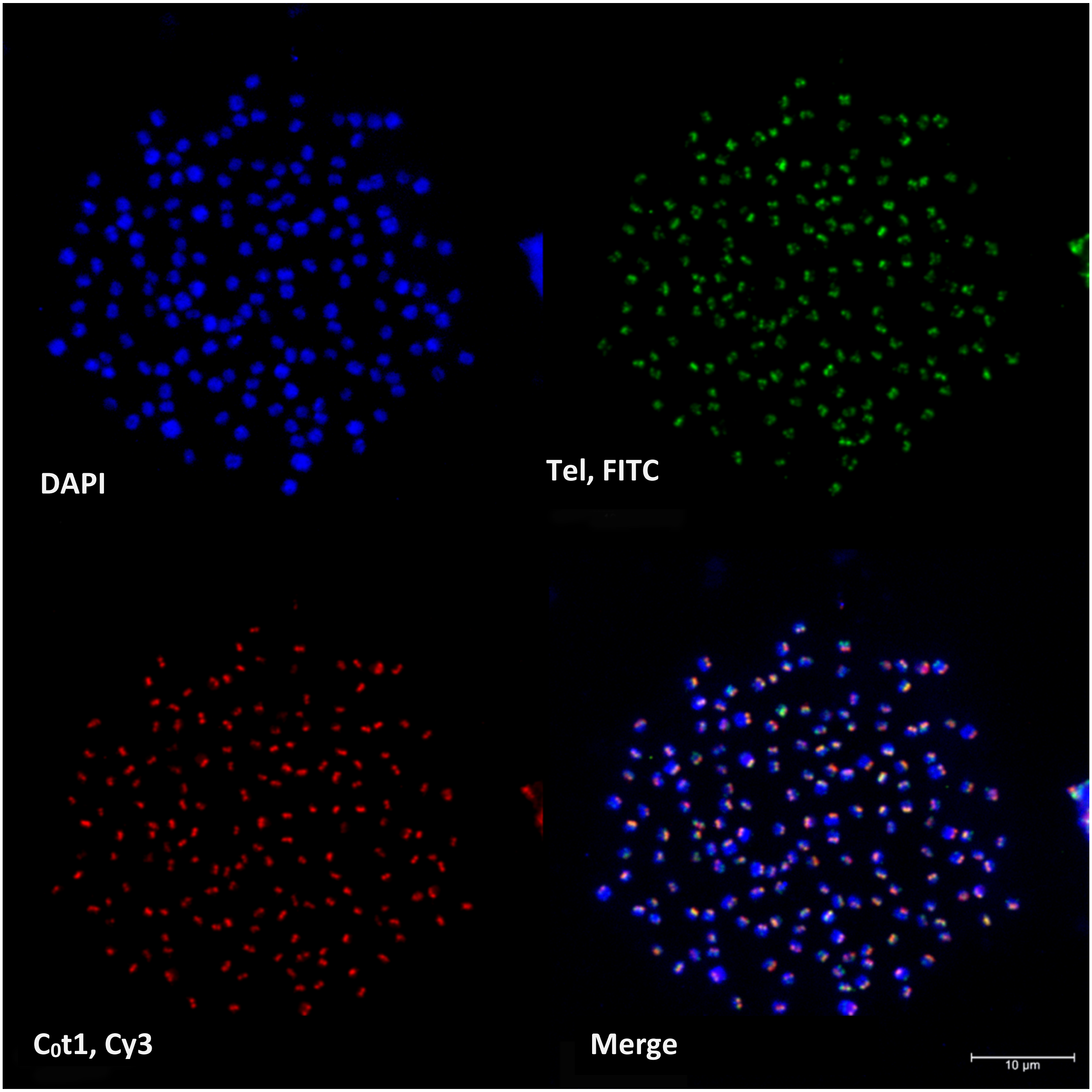

B.

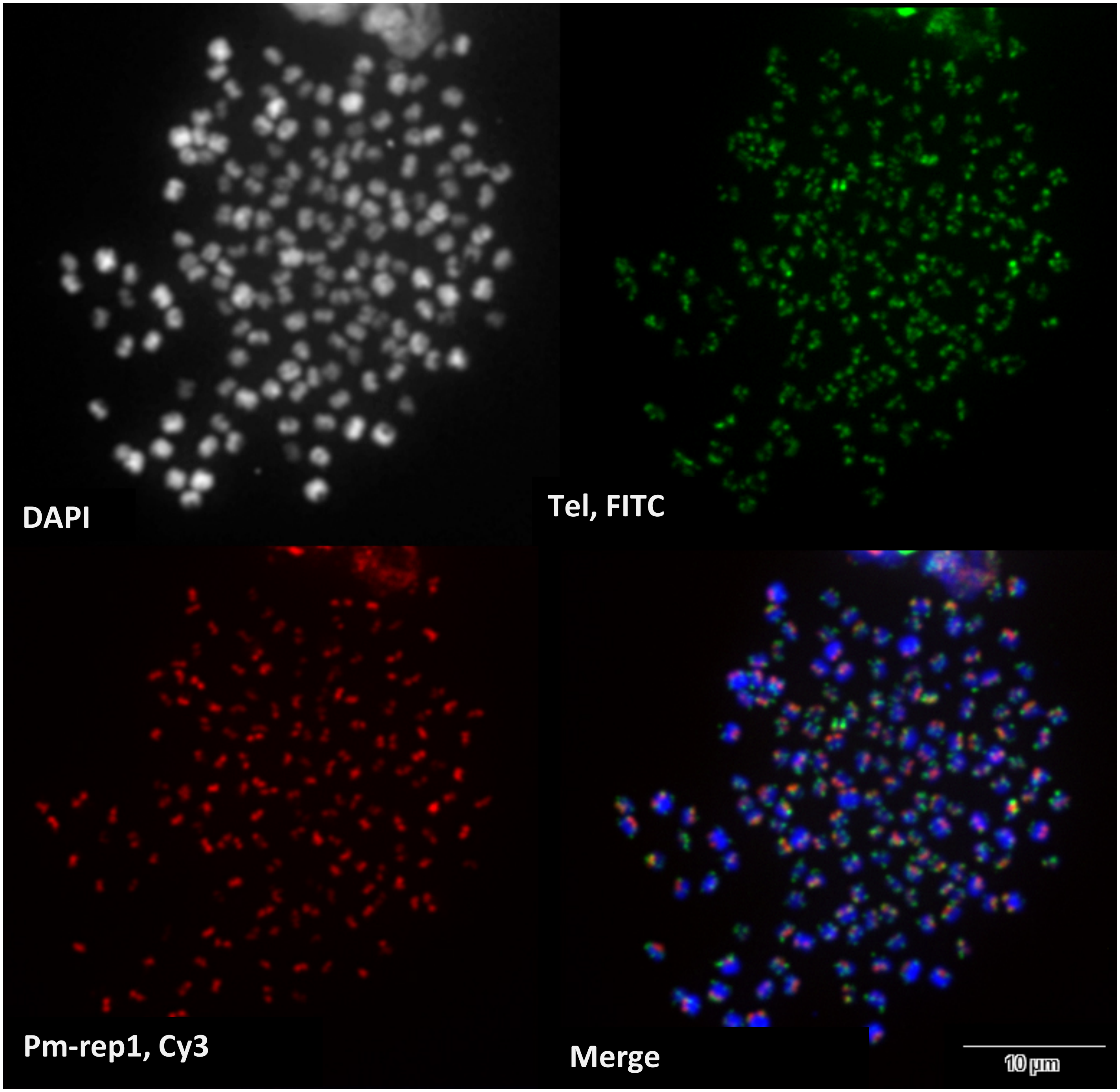

C.

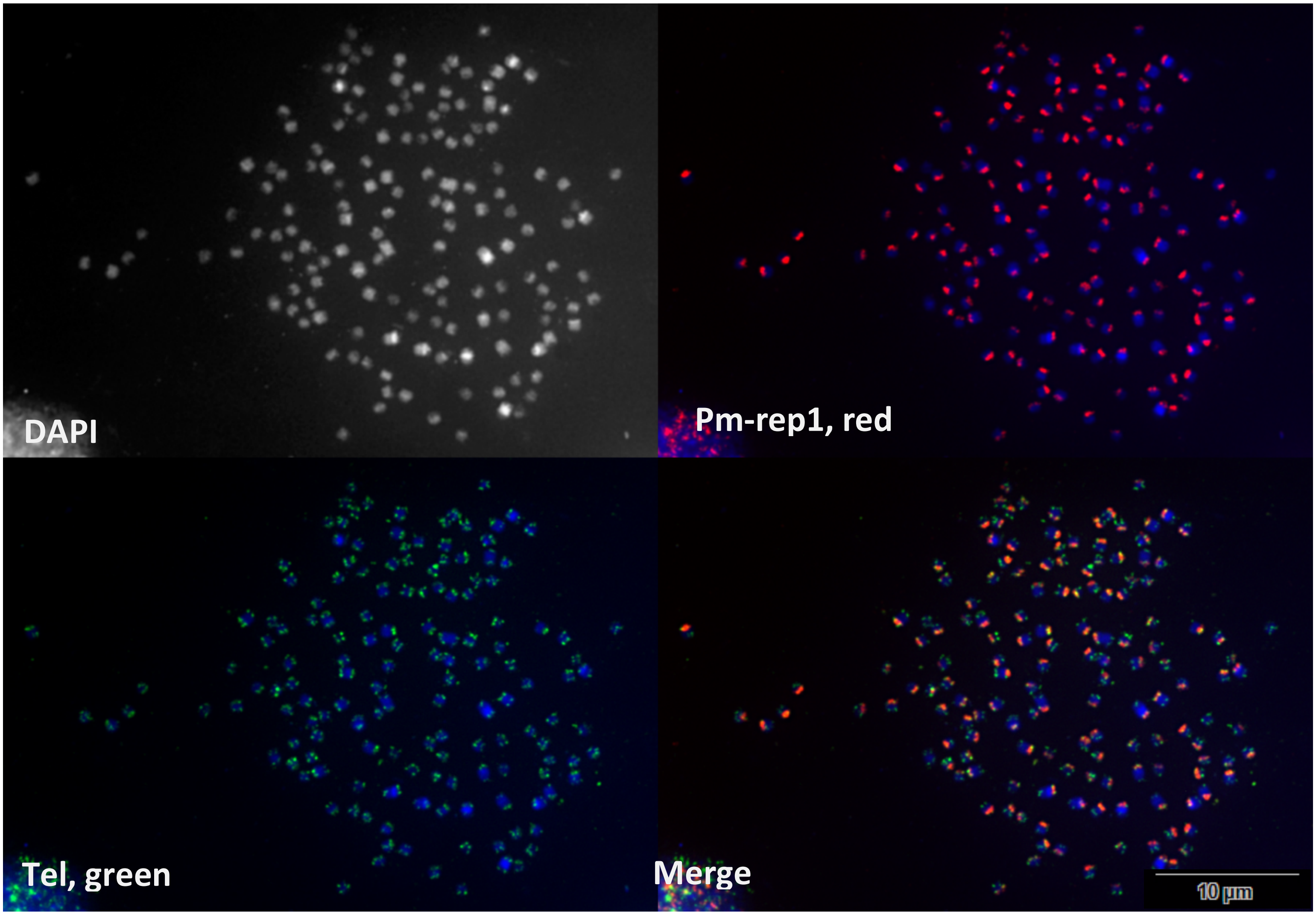

D.

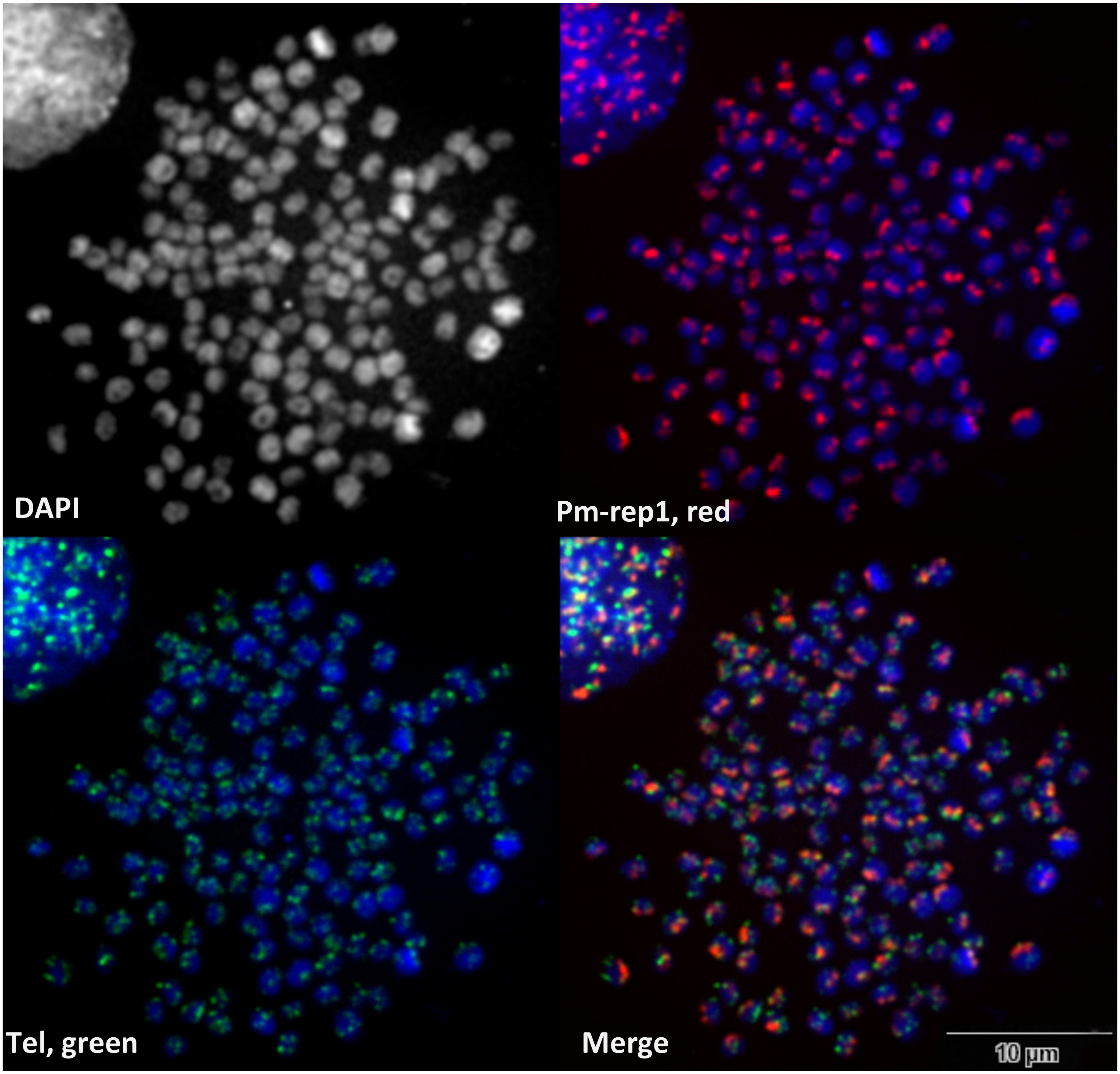

E.

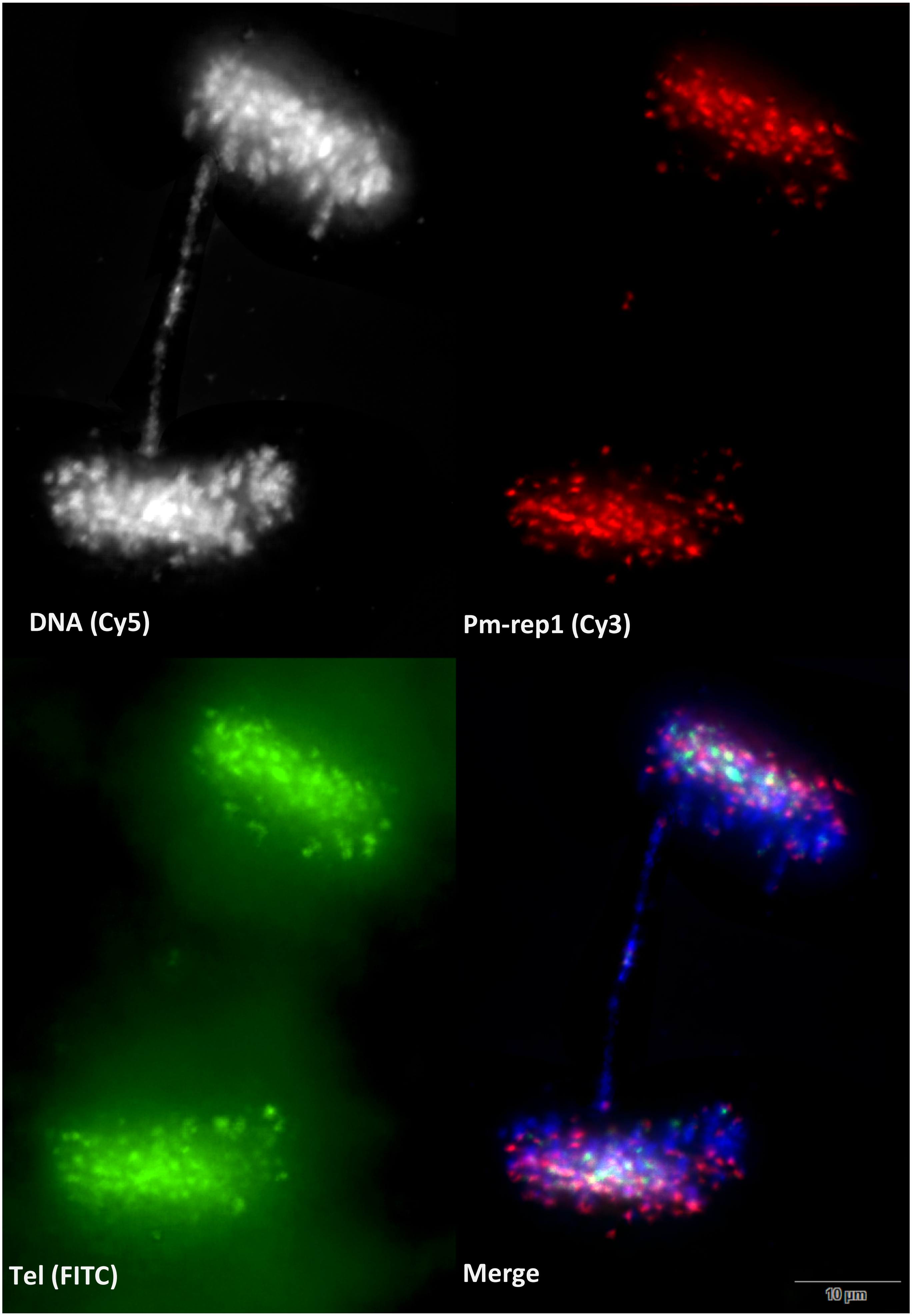

F.

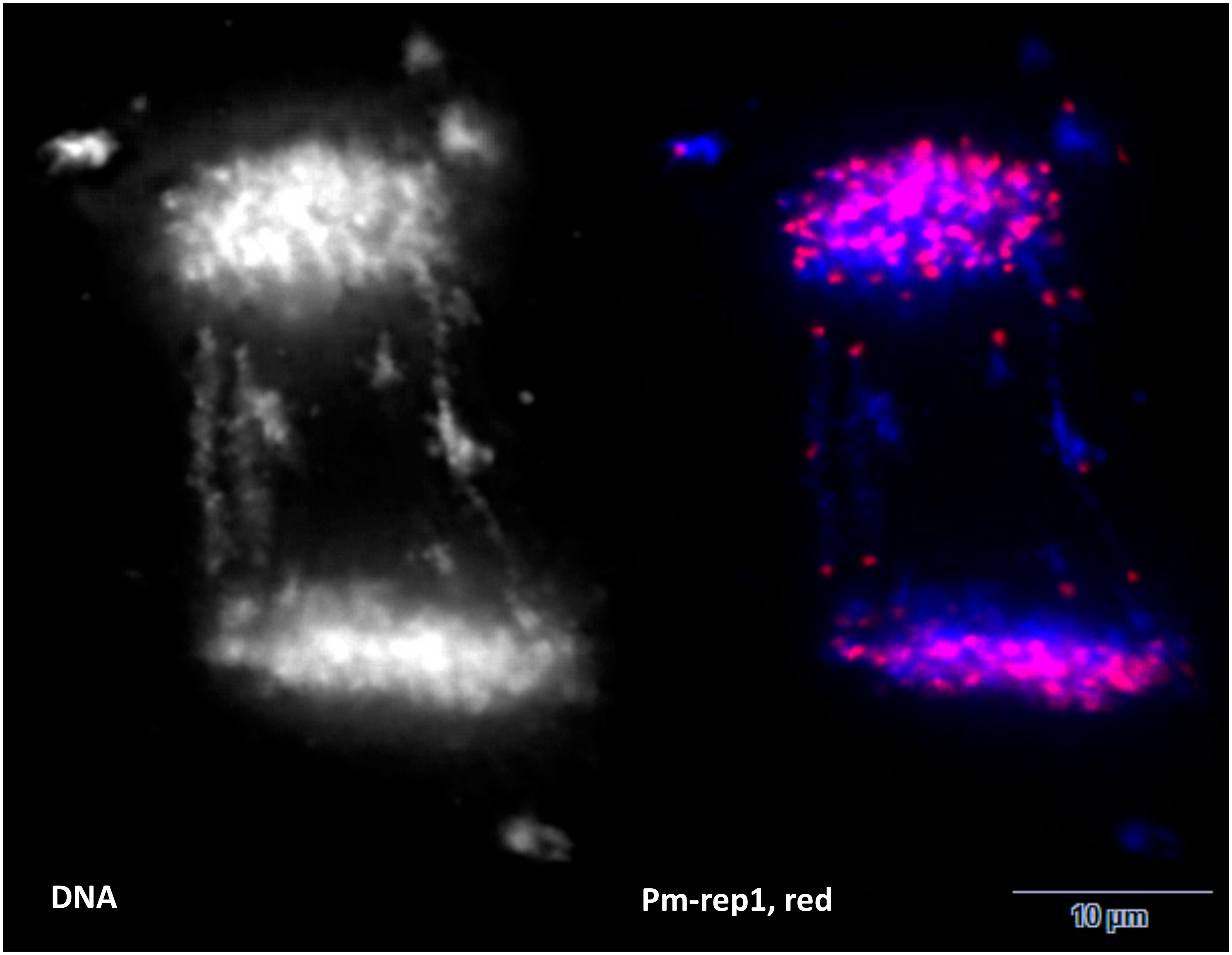

G.

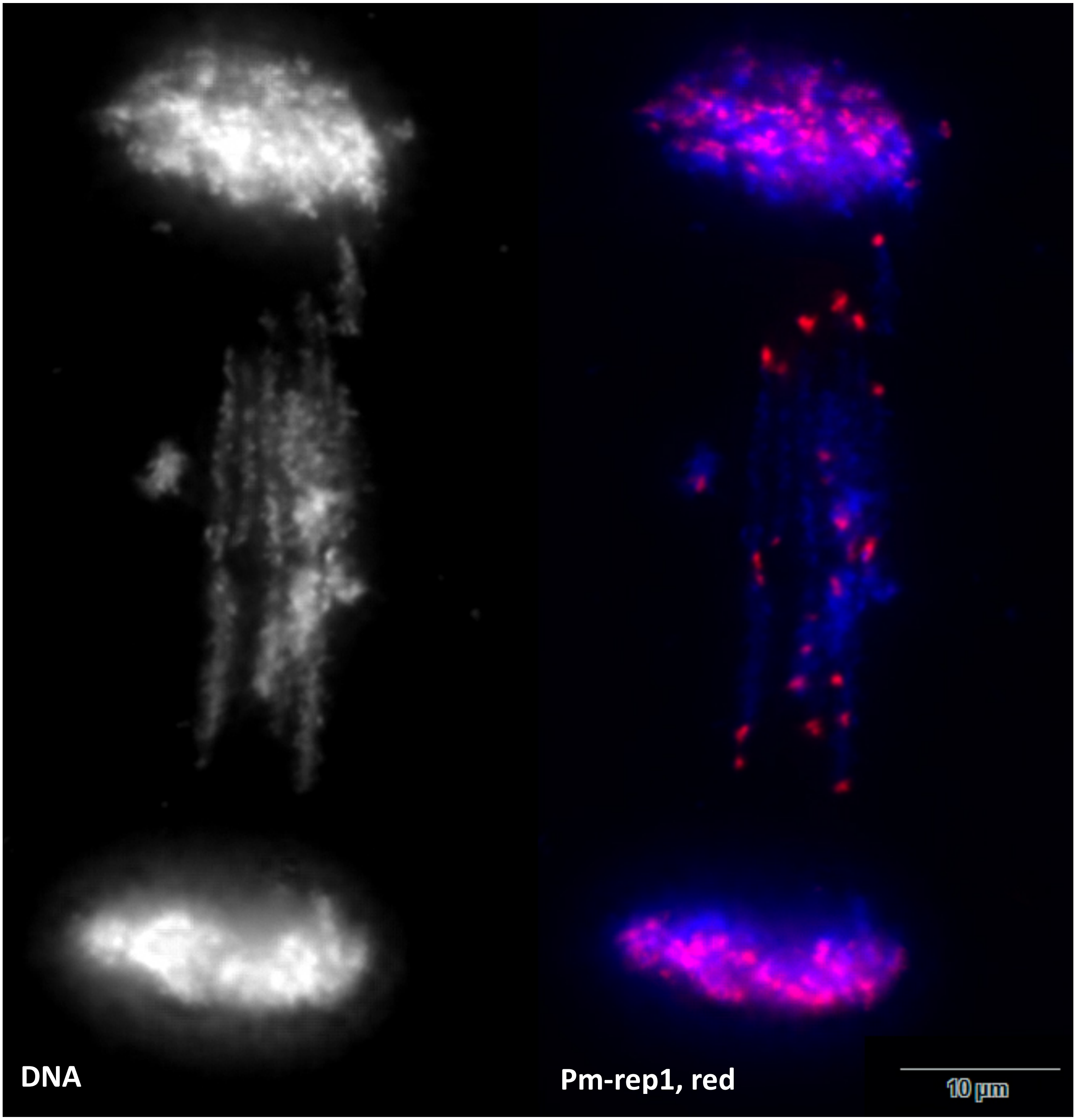

H.

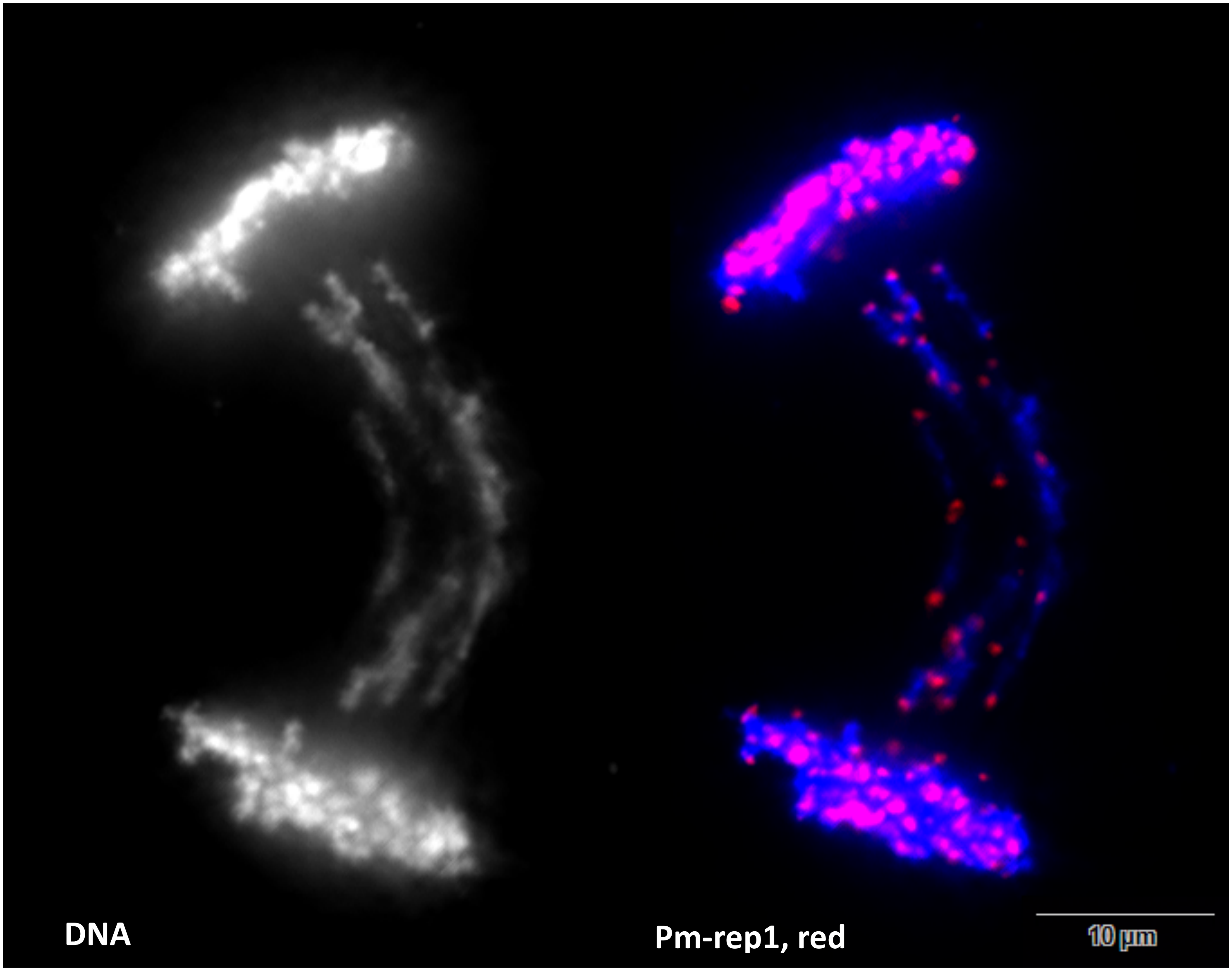

Figure S2. FISH of centromeric (*red*) and telomeric (*green*) probes on somatic spreads and lagging anaphases. The lamprey chromosomes are all small and appear to be acrocentric, having paired red centromeric signals on the one end of each chromosome. Note that chromatids are paired in mitotic metaphase. The green telomere signals mark both chromosome ends, however some chromosomes show clear differences in the strength of the telomeric signal at their two ends. (A) Cot1 (red) hybridization to a somatic spread. (B-D) Pm-rep1 (red) hybridization to a somatic spread. The Pm-rep1 probe shows specific centromeric signal for all somatically retained metaphase chromosomes of the sea lamprey. (E) Pm-rep1 (red) and telomeric PNA probe (green) to lagging anaphase 1.5 dfp embryo. Pm-rep1 signals appear on both edges of stretch chromosome overlapping with telomeric signals. (F-H) Pm-rep1 hybridization to lagging anaphases from 1.5 dpf embryos. Pm-rep1 signals localize to the poleward oriented end of both retained and lagging chromosomes. Weaker, presumably less specific, signals appear in the subtelomeric region distal to the centromeres of stretched chromosomes (near the original metaphase plane).
