# Supplementary material for: Germline-Specific Repetitive Elements in Programmatically Eliminated Chromosomes of the Sea Lamprey (Petromyzon marinus)
Source: Genes (Basel). 2019 Oct 22;10(10):832. doi: 10.3390/genes10100832 (PMC6826781; doi:10.3390/genes10100832)
Supplement: Supplementary file 1 [file genes-10-00832-s001.zip › Fig. S1.pdf]

Figure S1

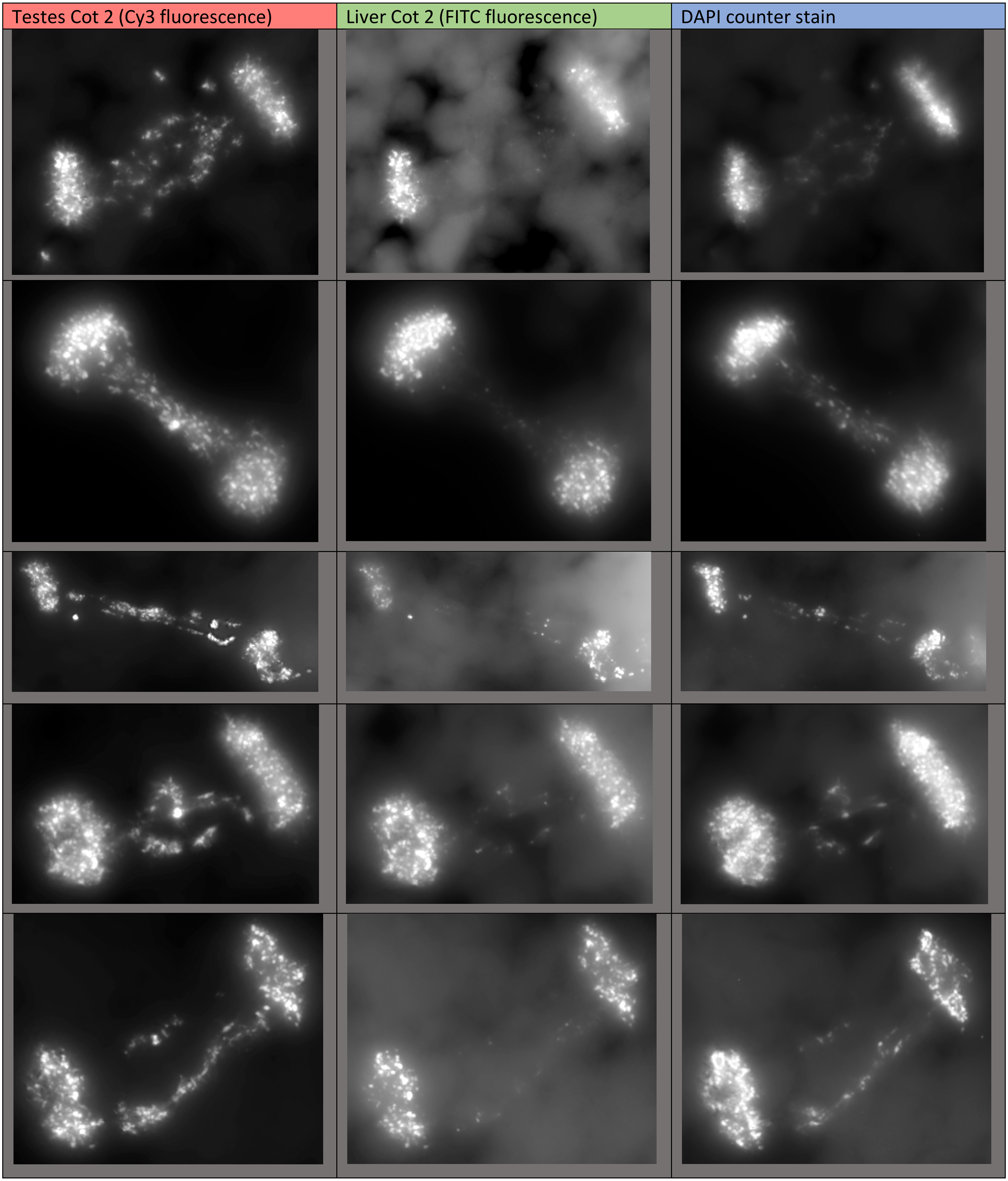
Testes Cot 2 (Cy3 fluorescence)
Liver Cot 2 (FITC fluorescence)
DAPI counter stain

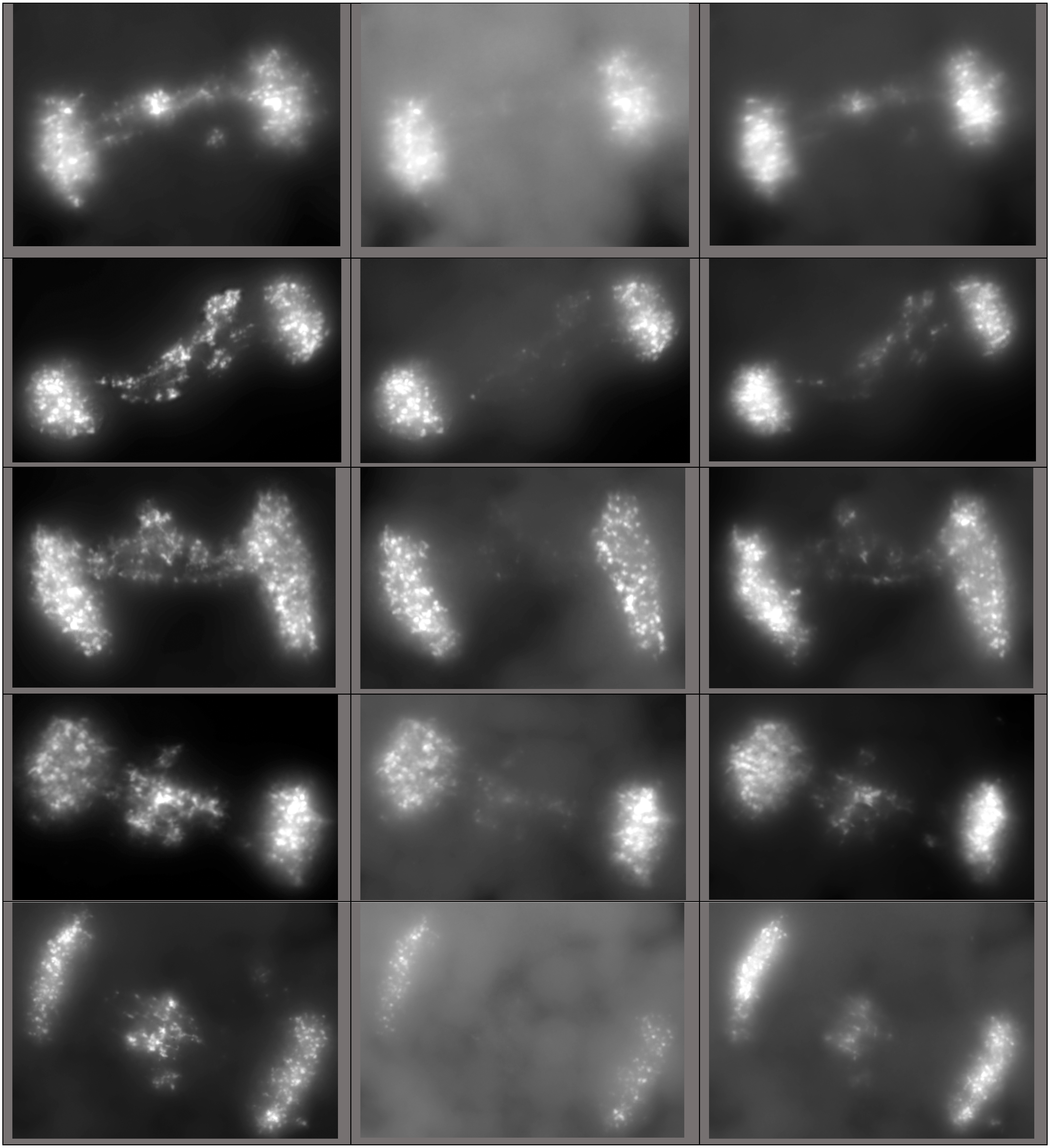

Figure S1. C_0t CGH on anaphase chromosomes of the sea lamprey embryos undergoing germline chromosome elimination. Germline repetitive DNA (C_0t2 isolated from testes) labels lagging chromosomes with equal or greater intensity in comparison to somatically retained chromosomes, whereas the fluorescence produced by a liver C_0t2 DNA probe is almost completely absent from lagging chromosomes but well represented in somatically retained chromosomes. Germline derived C_0t2-DNA labeled with Cy3 and somatic repetitive fraction labeled with FITC are shown in gray monochrome scale for better contrast.
